# Long-Lasting Exendin-4-Coated Gold Nanoparticles: Synthesis and In Vivo Evaluation of Hypoglycemic Activity

**DOI:** 10.3390/ph17111475

**Published:** 2024-11-02

**Authors:** Reeju Amatya, Amala Joseph, Gu Seob Roh, Yassmine Benmokadem, Kyoung Ah Min, Meong Cheol Shin

**Affiliations:** 1 College of Pharmacy and Research Institute of Pharmaceutical Sciences, Gyeongsang National University, 501 Jinju Daero, Jinju 52828, Gyeongnam, Republic of Korea; reejuamatya94@gmail.com (R.A.); amalakannen@gmail.com (A.J.); 2 Department of Anatomy and Convergence Medical Science, Metabolic Dysfunction Liver Disease Research Center, Institute of Medical Science, College of Medicine, Gyeongsang National University, 816-15 Jinju Daero, Jinju 52727, Gyeongnam, Republic of Korea; anaroh@gnu.ac.kr; 3 College of Pharmacy and Inje Institute of Pharmaceutical Sciences and Research, Inje University, 197 Injero, Gimhae 50834, Gyeongnam, Republic of Korea; yassmine.benmokadem@gmail.com

**Keywords:** exendin-4, gold, nanoparticle, diabetes, long-lasting

## Abstract

Background: Gold nanoparticles (NPs) have drawn great attention in the area of biomedical research with their relatively safe and versatile properties. This study aimed to synthesize long-lasting exendin-4-coated gold NPs (EX-ABD-AFF-GoldNPs) and evaluate their anti-diabetic effects in vivo. Methods: In the present study, EX-ABD-AFF-GoldNPs were synthesized using a simple one-step aqueous reduction method. The physical characterization of the prepared particles verified the successful formation of the EX-ABD-AFF-GoldNPs through dynamic light scattering (DLS), transmission electron microscopy (TEM), ultraviolet–visible (UV-VIS) light spectroscopy, and Fourier transform infrared spectroscopy (FTIR). The anti-hyperglycemic and anti-obesity effects were assessed in high-fat diet (HFD)-fed obese diabetic mice. Additionally, pharmacokinetics (PK) and biodistribution studies were performed to verify the long-lasting properties. Results: The EX-ABD-AFF-GoldNPs were conglomerates of smaller globular-shaped particles, and the average size was 110(±14) nm, based on the TEM images. Safety assessments using Min6, HepG2, and B16F10 cell lines demonstrated low cytotoxicity, with over 80% cell viability up to the highest tested concentration of 150 μg/mL (as EX-ABD-AFF). Notably, the animal studies showed that the EX-ABD-AFF-GoldNPs exhibited significant hypoglycemic activity, comparable to the EX-ABD-AFF, in the HFD-fed mice. A 4-week treatment with EX-ABD-AFF-GoldNPs produced similar reductions in blood glucose and body weight to the EX-ABD-AFF, without any apparent toxicity. Furthermore, the PK and biodistribution study results confirmed the long-lasting properties (plasma half-life: 43.6 h) of the particles. Conclusions: Overall, this study demonstrated that the preparation of therapeutic protein-loaded gold NPs is feasible and, despite their much larger size compared with the protein, EX-ABD-AFF-GoldNPs can be successfully absorbed through the subcutaneous route and show nearly equivalent hypoglycemic activity to the EX-ABD-AFF protein. Finally, this study showed that long-lasting properties could be acquired by only coating EX-ABD-AFF onto gold NPs.

## 1. Introduction

Metal NPs, such as gold NPs, have drawn great interest in various biomedical research fields [1,2]. Gold NPs exhibit low toxicity as drug carriers, making them safe for use in biological systems [3]. Because of their unique rigid structure, different from those of lipid-based or virus-based NPs, they can be specifically appropriate for protein delivery [4,5,6]. Their tunable size and easily modifiable surface facilitate the attachment of various therapeutic agents, targeting ligands and stabilizing molecules, thereby enhancing their functionality [7]. Furthermore, their plasmonic properties allow gold NPs to be suitable for imaging and diagnostics [8]. Many researchers first found their applicability for cancer therapy, because of their low toxicity as drug carriers, tunable size, feasible surface modification, and plasmonic nature [8,9,10,11,12]. Their photothermal nature also enables them to convert light energy into heat, providing an effective cancer treatment method by destroying cancer cells through localized hyperthermia. These properties make gold NPs highly versatile and promising tools in the field of oncology [8,9,10,11,12,13]. To utilize gold NPs as a nanoscale drug delivery system in cancer therapy, various natural compounds, such as curcumin [14], and small molecules, such as doxorubicin [4,15], have been incorporated into gold NPs to achieve effective targeted delivery. However, these characteristics of gold NPs may also be exploited in different medical fields, such as the treatment of diabetes [16,17].

Yet, to date, successful macromolecule nanoencapsulation has been a formidable task, because it requires aqueous and ambient synthesis conditions [18,19]. Furthermore, even if the loading is successful, the release of the drugs used is severely impeded due to their large size [20]. In this regard, drug adsorption on the surfaces of metal NPs could serve as an effective alternative approach [21,22,23,24]. One of the most widely adopted methods for synthesizing metal NPs is reducing the metal ions in the presence of hydrophilic coating materials [25,26,27]. Due to hydrophobicity, reduced metal elements tend to form aggregates, and the adjacent coating materials spontaneously adsorb onto the metal surface (generally via Van der Waals force) stabilizing the particles. Many different types of coating materials have been used, which include albumin proteins [28,29,30]. As the reduction process could occur in aqueous and ambient temperatures, not only albumin, but possibly various protein drugs may be loaded onto metal NPs via physical adsorption [31,32]. Peptide hormones, such as insulin, have been successfully loaded onto gold NPs, resulting in improved delivery and enhanced hypoglycemic effects [33]. One of the key factors influencing protein adsorption onto NPs is their surface area. NPs with a higher surface-to-volume ratio offer an increased surface area for protein binding, enabling greater protein adsorption irrespective of mass. This characteristic makes gold NPs particularly intriguing [34].

EX-ABD-AFF is a long-acting exendin-4 fusion protein (molecular weight: 31.2 kDa), previously developed in our laboratory [35,36]. An albumin binding domain (ABD) and an anti-FcRn affibody (AFF) are tandem-fused to the exendin-4, enabling efficient binding to plasma albumin (which enlarges the size of the protein, sufficient to impede the glomerular filtration), as well as effective FcRn-mediated recycling [35]. Due to these combined effects, the plasma half-life of exendin-4 was extended to about ~7 days (markedly increased compared with the 0.7 h of the unmodified exendin-4), and the hypoglycemic effects lasted for more than 12 days [35]. These significantly extended therapeutic effects make the EX-ABD-AFF a favorable diabetic therapeutic and also a great model drug to test the NP-based drug delivery system.

In this study, we developed EX-ABD-AFF-GoldNPs and evaluated their pharmacokinetics (PK), biodistribution profiles, and treatment efficacy for diabetes. The EX-ABD-AFF-GoldNPs were synthesized via a simple one-step reduction process, and the prepared NPs were assessed for physical properties and in vivo efficacy against diabetes. Despite the large size of the particles, the EX-ABD-AFF-GoldNPs could be successfully absorbed through the subcutaneous route and showed nearly equivalent hypoglycemic activity to the EX-ABD-AFF protein.

## 2. Results and Discussion

### 2.1. Synthesis and Physical Characterization of EX-ABD-AFF-GoldNPs

To date, various synthesis methods of gold NPs have been devised, and the reduction method in aqueous solution has been widely used for particle preparations [37,38]. This is due to the merits of simple and easy procedures and satisfactory yield. To synthesize gold NPs, the reduction reaction is typically carried out at temperatures above 60 °C. However, when adjusting the experimental conditions, the ambient temperature may also allow the synthesis of gold NPs, and this may enable the preparation of NPs coated with functional protein drugs.

**Figure 1:**
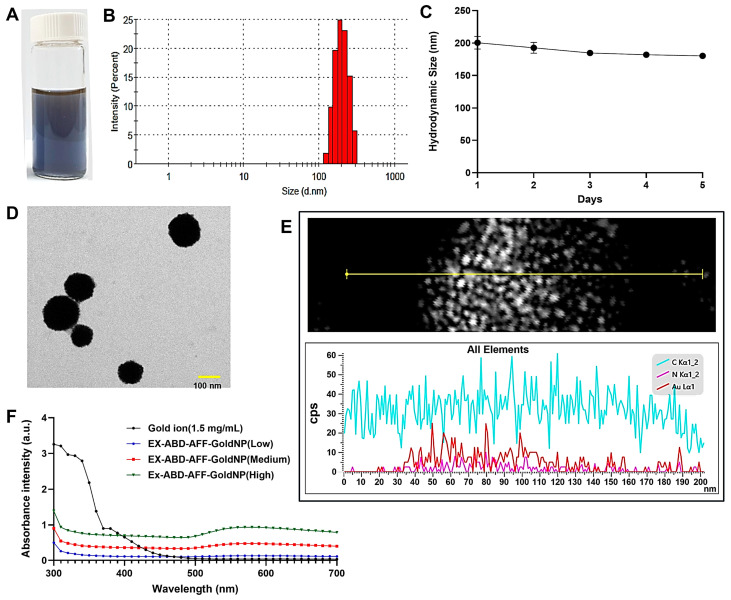
Physical characterization of EX-ABD-AFF-GoldNPs. (**A**) Image of the EX-ABD-AFF-GoldNP suspension. (**B**) Hydrodynamic size distribution and (**C**) size stability by DLS. (**D**) BIO-TEM image of EX-ABD-AFF-GoldNPs, (**E**) High-resolution transmission electron microscopic (HR-TEM) image of the particles and elemental distribution of carbon (C), nitrogen (N), and gold (Au) in the TEM particle images. (**F**) UV-VIS spectrum of EX-ABD-AFF-GoldNPs (EX-ABD-AFF-GoldNPs: EX-ABD-AFF-coated gold nanoparticles).

Here, the EX-ABD-AFF-GoldNPs were prepared by a one-step reduction at RT in an aqueous solution. In the colorless mixture of EX-ABD-AFF and ascorbic acid (reducing agent), the addition of gold ions changed the solution to dark blue without causing any apparent aggregation (Figure 1A). After three runs of centrifugation with an ultracentrifugal device (membrane pore size: 100 kDa), the unbound EX-ABD-AFF could be successfully removed. The final composition of the EX-ABD-AFF-GoldNPs was found to contain an average of 1.2 mg of gold and 610 μg of EX-ABD-AFF.

The average hydrodynamic size of the EX-ABD-AFF-GoldNPs was 183 (±21) nm (PDI: 0.15), which was larger than the average size (110 (±14) nm) observed from the BIO-TEM images (Figure 1B). At the storage condition of 4 °C, the particle samples retained their initial size for 5 days, suggesting good size stability, sufficient to prepare once weekly for further animal studies (Figure 1C). In the Bio-TEM images, the shape of the EX-ABD-AFF-GoldNPs appeared globular (Figure 1D). Interestingly, the HR-TEM showed that the 100 nm particles observed from the BIO-TEM image were actually much smaller particles (5–10 nm in diameter) conglomerating together. The elemental analysis results evidenced the presence of gold and protein in the particles (Figure 1E). The mean zeta potential was −21 (±6.3) mV (measured at pH 7.4 in deionized water (DW) by DLS), reflecting the negatively charged protein coating on the surfaces of the particles.

**Figure 2:**
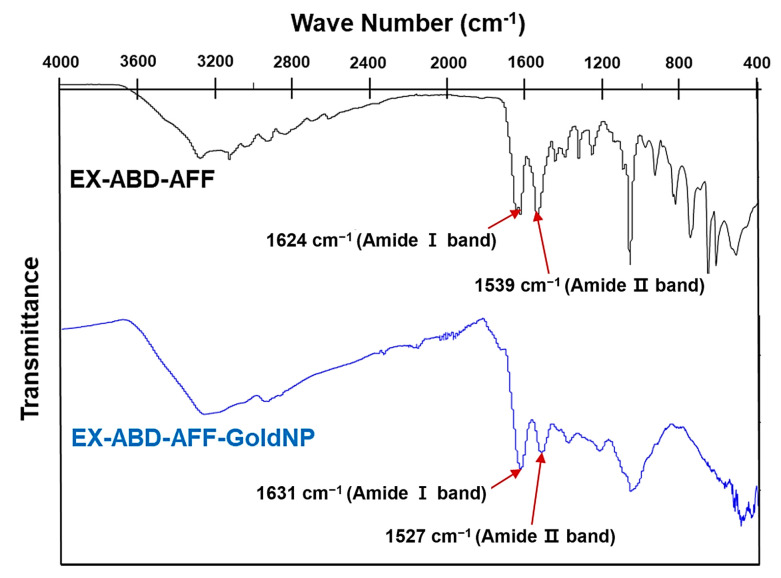
Fourier transform infrared (FT-IR) spectroscopic profiles of EX-ABD-AFF-GoldNPs.

To finally verify the synthesis of the EX-ABD-AFF-GoldNPs, the UV-VIS spectra of gold ion and difference concentrations of the EX-ABD-AFF-GoldNPs were compared (Figure 1F). The UV-VIS spectra can be useful in determining the sizes and shapes of gold NPs [39]. Gold NPs exhibit a characteristic strong absorbance band in the 500–600 nm range due to their localized surface plasmon resonance (LSPR) [39]. It was shown that the EX-ABD-AFF-GoldNPs had markedly differential spectra from the gold ion. While the gold ion showed high intensity at 300–400 nm [40,41], the spectra of the particles had relatively high intensity at 500–600 nm. Moreover, the observed outcome aligns with reports showing a correlation between the absorption peak wavelength and particle diameter [42,43]. Furthermore, from the FT-IR results (Figure 2), the peaks of the EX-ABD-AFF protein (at 1624 and 1539 cm^−1^, attributed to the bands of amide I and II) could have appeared in the spectrum of the EX-ABD-AFF-GoldNPs (1631 and 1527 cm^−1^). Overall, all the results evidenced the successful synthesis of the EX-ABD-AFF-GoldNPs [44].

### 2.2. Cytotoxicity of EX-ABD-AFF-GoldNPs

**Figure 3:**
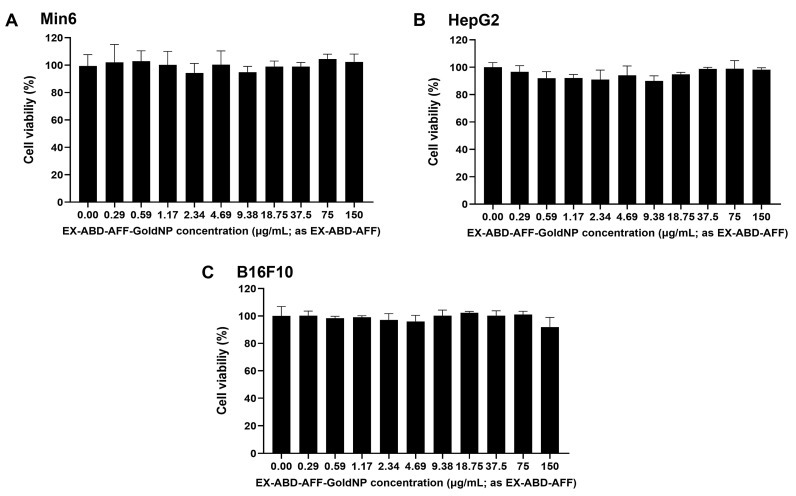
Cytotoxicity profile of EX-ABD-AFF-GoldNPs for (**A**) Min6, (**B**) HepG2, and (**C**) B16F10 cells.

The cytotoxicity of the EX-ABD-AFF-GoldNPs was assessed with different relevant cell lines (Min6, HepG2, and B16F10). The selection of these cells was based on the major absorption site (skin; B16F10) [45], potential major elimination site (liver; HepG2), and potential action site (pancreas; Min6) [46,47]. The cytotoxicity assay results are shown in Figure 3. For all the tested cell lines, the EX-ABD-AFF-GoldNP showed little toxicity up to the highest tested concentration of 150 μg/mL (as gold). Overall, the results evidenced the safety of the in vivo application of the particles. However, these results should be interpreted with good care. Because of the poor biodegradability of the gold, like other metal-based NPs, gold NPs may not be free from long-term toxicity or unexpected immune responses.

### 2.3. GTT

**Figure 4:**
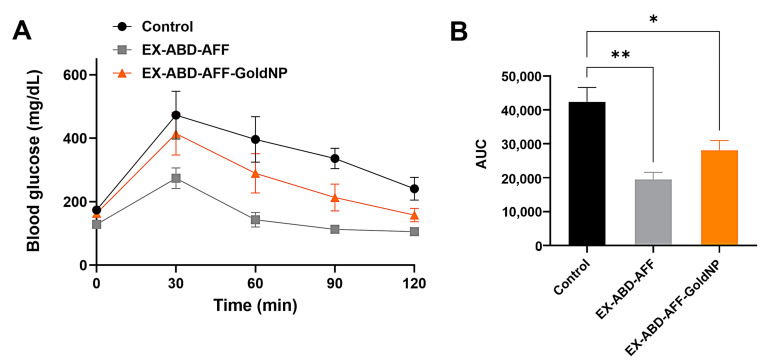
Glucose tolerance test (GTT) results in high-fat-diet-fed C57BL/6 obese mice. (**A**) Blood glucose level vs. time profile after subcutaneous injection of EX-ABD-AFF-GoldNPs and glucose. (**B**) Comparison of the area under the curves (AUCs). The statistically significant differences among the groups were compared by one-way ANOVA (Tukey’s multiple comparison test as the post hoc test). * *p* < 0.05, ** *p* < 0.01.

A GTT assay was performed with the 12-week-HFD-fed obese and diabetic mice [48]. As shown in Figure 4A,B, compared with the control group, significantly lower increases in the blood glucose levels were observed from both the EX-ABD-AFF and EX-ABD-AFF-GoldNP groups.

### 2.4. Single-Administration Efficacy Study

**Figure 5:**
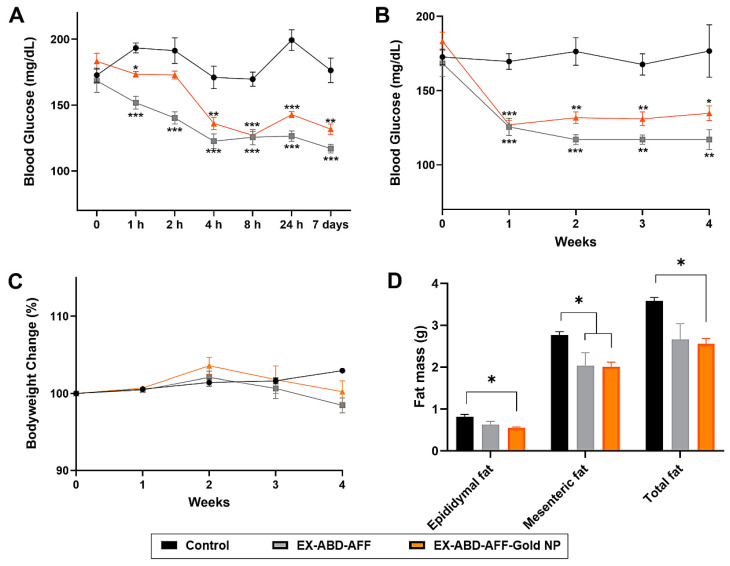
Hypoglycemic efficacy study results in high-fat-diet-fed C57BL/6 obese mice. (**A**) Blood glucose levels along the time after a single administration of EX-ABD-AFF-GoldNP. The mice’s blood glucose level profiles (**B**) and body weight changes (**C**) during the 4-week long-term efficacy study (twice-weekly administration of EX-ABD-AFF-GoldNPs). (**D**) Average fat weights of the mice after termination of the long-term study. The statistically significant differences among the groups were compared by one-way ANOVA (Tukey’s multiple comparison test as the post hoc test). * *p* < 0.05, ** *p* < 0.01, *** *p* < 0.001.

With the HFD-fed obese and diabetic mice, the treatment with both the EX-ABD-AFF and the EX-ABD-AFF-GoldNP provided significant hypoglycemic efficacy, which lasted for more than a week (Figure 5A), in good accordance with our previous report for the EX-ABD-AFF [35]. Based on the cytotoxicity study results (little toxicity up to 150 μg/mL), as expected, no signs of toxicity were observed at the 50 nmol/kg dose (90 μg/mouse). Assuming about 3 mL of total plasma volume for a mouse [49], the maximum concentration of 30 μg/mL (as protein) of EX-ABD-AFF-GoldNPs would be harmless to the animals. Consistent with the slower absorption of the EX-ABD-AFF-GoldNPs (C_max_: 8 h) than the EX-ABD-AFF, the induction of the hypoglycemic effects took a longer time. However, the effects similarly lasted up to a week.

### 2.5. Long-Term (4-Week) Efficacy

The other groups of HFD-fed C57/BL6 mice were allocated into three groups (five mice for each group): (1) control, (2) EX-ABD-AFF, and (3) EX-ABD-AFF-GoldNP. The control mice were injected with saline, while the other groups were administered via s.c. with EX-ABD-AFF at 50 nmol/kg and EX-ABD-AFF-GoldNP (50 nmol/kg as EX-ABD-AFF). As shown in Figure 5B,C, the EX-ABD-AFF-GoldNPs revealed similar-to-equivalent effects on blood glucose lowering and body weight loss. Notably, fat loss was observed in both treatment groups, but the effect was more significant in the EX-ABD-AFF-GoldNP-treated group (Figure 5D). During the study, specifically, for the EX-ABD-AFF-GoldNP-treated group, after repeated injections, slight inflammation was observed in the skin of the injection site, but no obvious signs of toxicity were observed. Regarding the nanotoxicity issues, it appears that gold NPs have high LD_50_ values above 5000 mg/kg, but the tissue distribution profiles and toxicity levels could differ depending on species and particle sizes [50]. Interestingly, compared with mice, rats showed higher gold NP accumulation in the spleen and, notably, the brain, which implies that a portion of the administered gold NPs may be able to penetrate through the blood–brain barrier [51,52,53]. Further examination would be required to confirm whether the EX-ABD-AFF-GoldNPs could also enter the brain, like the EX-ABD-AFF [35].

### 2.6. PK and Biodistribution Study

**Figure 6:**
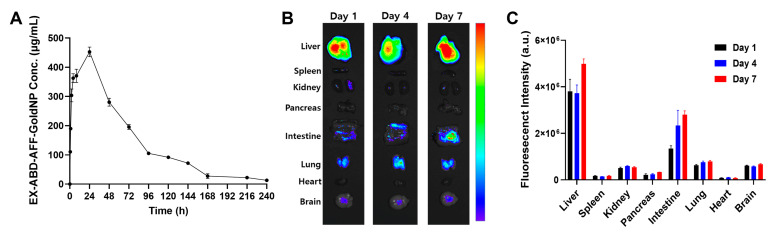
PK and biodistribution profiles of EX-ABD-AFF-GoldNPs. (**A**) Plasma-concentration-versus-time profile of RITC-labeled EX-ABD-AFF-GoldNPs after subcutaneous injection in C57BL/6 mice (*n* = 3). (**B**) Representative mouse organ images after subcutaneous administration of RITC-labeled-EX-ABD-AFF-GoldNPs. (**C**) Fluorescent intensity profiles of RITC-labeled-EX-ABD-AFF-GoldNPs in major organs at days 1, 4, and 7 post-administration (RITC: rhodamine B isothiocyanate).

To confirm the long-lasting properties of the EX-ABD-AFF-GoldNPs, the PK profiles were acquired using fluorimetry. As shown in Figure 6A, the particles were traceable in the plasma up to 10 days after administration. Notably, based on the PK analyses, the average plasma half-life was 43.6 h and the mean residence time was as long as 65.1 h. The biodistribution results (Figure 6B,C) correlated well with the PK results. Even on day 7 post-administration, strong fluorescence signals could be observed in the major organs (especially the liver). As the liver generally serves as the major elimination site for particles, these results showed promise in that it may be possible to further utilize EX-ABD-AFF-GoldNPs as drug carriers targeting the liver for diabetes-related metabolic disorders, such as non-alcoholic fatty liver disease.

## 3. Materials and Methods

### 3.1. Materials

BL21 *E.coli* was obtained from the Real Biotech Co. (Taipei, Taiwan). Isopropyl-β-thiogalactopyranoside (IPTG) and LB broth were obtained from Thermo Fisher Scientific (Waltham, MA, USA). Kanamycin, ascorbic acid, and gold (III) chloride trihydrate (HAuCl_4_·3H_2_O) were purchased from Sigma Aldrich (St. Louis, MO, USA). All the chemicals used were analytical-grade.

### 3.2. Production of EX-ABD-AFF

The EX-ABD-AFF fusion protein was produced following the established method described by Lee et al. [35]. Briefly, one colony of BL21 *E. coli* transformed with pET28a-SUMO-EX-ABD-AFF was added to 50 mL of LB medium with 80 μg/mL of kanamycin. Incubation of the starter culture was performed with shaking at 250 rpm at 37 °C overnight, and then 1 L of LB medium with 80 μg/mL of kanamycin was added to the culture for dilution. The 1 L culture was incubated again with a shaking condition at 37 °C overnight. When the optical density at the wavelength of 600 nm reached about 1, IPTG was added to become 0.5 mM as a final concentration. The culture was incubated for 4 h, and then the cell cultures were centrifuged for the cell harvesting. The cells were dispersed in PBS with 20 mM phosphate buffer (pH 7) containing 300 mM NaCl, and lysed using a sonicator (30-s run and 30-s break for 3 cycles). After the centrifugation of the lysed *E. coli* cells, the supernatant was collected and loaded onto Talon superflow resin (GE Healthcare Bio-Sciences, Pittsburgh, PA, USA). The EX-ABD-AFF was purified and concentrated using Amicon^®^ Ultra-15 Centrifugal Filters (MW cut-off: 30 kDa, Merck KGaA, Darmstadt, Germany). The samples were centrifuged at 3000 rpm for 15 min for a total of 3 cycles. The purified protein was stored at 4 °C refrigerator until use.

### 3.3. EX-ABD-AFF-GoldNP Synthesis

EX-ABD-AFF 3 mL (1 mg/mL in deionized water (DW)) and ascorbic acid (reducing agent) 5 mL (53 mg/mL in DW) were mixed and stirred at 650 rpm at room temperature (RT) for 10 min. Next, 100 µL of 30 mg/mL gold (III) chloride trihydrate were added dropwise using a syringe (30-gauge needle) and the reaction mixture was further stirred at 650 rpm for 20 min at RT. The colorless solution turned into a dark blue color, indicating the formation of gold NPs. The ascorbic acid and unbound EX-ABD-AFF were removed by using an ultra-centrifugal device with Amicon^®^ Ultra-15 centrifugal filters (membrane cutoff: 100 kDa, Merck Millipore, Darmstadt, Germany). The volume of the final EX-ABD-AFF-GoldNP sample was adjusted to 1 mL. The EX-ABD-AFF bound on the particles was quantified by micro-Bradford assay. The gold content was calculated by the measurement of the inductively coupled plasma mass spectrometry (ICP-MS).

### 3.4. Physical Characterization

The size and morphology of the EX-ABD-AFF-GoldNPs were analyzed using Tecnai 12 FEI Bio 120 kV transmission electron microscopy (Hillsboro, OR, USA). The morphology and composition of the particles were further studied using high-resolution 300 kV field-emission TEM with energy-dispersive spectroscopy (Tecnai TF30 ST HR-TEM/EDS, FEI Co., Hillsboro, OR, USA). The absorption spectra in the UV-VIS wavelength range of 300–800 nm were measured using a Synergy H1 plate reader (BioTek Instruments, Winooski, VT, USA). The NPs’ size and zeta potential were identified with the dynamic light scattering (DLS) of Zetasizer Nano ZS (Malvern Panalytical Ltd., Malvern, UK). The zeta potential was measured by a zeta sizer based on the Gouy–Chapman–Stern theory. The size stability of EX-ABD-AFF-GoldNPs (at 4 °C) was assessed by measuring the particle size for 5 consecutive days. The FT-IR was carried out using an FT-IR micro-spectrometer (VERTEX 80v, Bruker, Billerica, MA, USA).

### 3.5. Cell Viability Assay

The cytotoxicity of the EX-ABD-AFF-GoldNPs was examined on various cell lines (e.g., Min6, HepG2, and B16F10). Min6 (mouse insulinoma β cells) was kindly provided by Dr. Min Gap Kim (Gyeongsang National University, Jinju-si, Republic of Korea) [54,55]. B16F10 (mouse melanoma; ATCC-CRL-6475™) cells were obtained from ATCC (Manassas, VA, USA) and HepG2 (human hepatoblastoma; KCLB No. 88065) cells from Korean Cell Line Bank (Seoul, Republic of Korea). All the cells were cultured in Dulbecco’s Modified Eagle Medium (DMEM), containing 10% fetal bovine serum (FBS), 1% penicillin–streptomycin, and 1% antibiotic–antimycotic. The cells (1 × 10^4^ cells/well) were separately seeded onto 96-well plates for the cell viability assay. After overnight incubation in the humidified incubator at 37 °C under 5% CO_2_, the EX-ABD-AFF-GoldNP sample was treated with the cells at varying concentrations (0–150 μg/mL as EX-ABD-AFF). After 48 h incubation of the cells, the WST-1 assay kit (iNtRON Biotechnology, Daejeon, Republic of Korea) was used to assess the cell viability.

### 3.6. Animal Studies

The animal studies were carried out following the protocol approved by the GNU’s Committee for Animal Research (GNU-230615-M0131), in compliance with the National Institute of Health (NIH) Guidelines on the Use of Laboratory Animals.

#### 3.6.1. Glucose Tolerance Test (GTT)

The GTT was carried out after fasting the mice for 16 h. Each treatment was performed for the mouse group as either saline (control), EX-ABD-AFF (50 nmol/kg), or EX-ABD-AFF-GoldNP (50 nmol/kg as EX-ABD-AFF) by s.c. injection, and then 1 h later, challenged with i.p. injection of D-glucose (2 g/kg; Sigma-Aldrich, St. Louis, MO, USA). A small volume of blood was withdrawn at 0, 30, 60, 90, and 120 min post-administration of the D-glucose, and the glucose levels were confirmed using an Accu-Chek glucometer (Roche Diagnostics GmbH, Mannheim, Germany).

#### 3.6.2. Single-Administration Study

To assess the functionality of the samples, C57BL/6 obese mice were administered 12 weeks of high-fat diet (HFD) (60% kcal fat; 5.24 kcal/g, Research Diets, New Brunswick, NJ, USA). The 3-week-old C57BL/6 mice in three groups (*n* = 5) were administered (via s.c.) either saline, EX-ABD-AFF (50 nmol/kg), or EX-ABD-AFF-GoldNP (50 nmol/kg as EX-ABD-AFF). After the sample injection, the blood glucose level was monitored for the following 7 days.

#### 3.6.3. Long-Term (4-Week) Efficacy Study

The 12-week-HFD-fed obese C57BL/6 mice were allocated into three groups (5 mice for each group): (1) control, (2) EX-ABD-AFF, and (3) EX-ABD-AFF-GoldNP. The control mice were administered with saline, and the EX-ABD-AFF and EX-ABD-AFF-GoldNP groups were treated with EX-ABD-AFF and EX-ABD-AFF-GoldNPs (50 nmol/kg, as with EX-ABD-AFF), respectively. The treatment was carried out once a week for 4 weeks via s.c. injection. During the study, the mice’s body weights and blood glucose levels were monitored. After the termination of the study, mice were euthanized and the fats (epididymal fat and mesenteric fat) were collected and weighed.

#### 3.6.4. PK

Prior to the PK study, the EX-ABD-AFF-GoldNPs were labeled with rhodamine (RITC), a fluorescent dye. In a glass vial, 1 mL of EX-ABD-AFF (5 mg/mL) was mixed with 100 µL of RITC (2.5 mg/mL in DMSO). The mixture was kept at room temperature (RT) for 2 h with gentle stirring in the dark. After incubation, the RITC-labeled EX-ABD-AFF was purified using Talon affinity resins, followed by further purification using an ultra-centrifugal filter unit (MWCO: 30 kDa). The final sample volume was adjusted to 1 mL. The RITC-labeled EX-ABD-AFF-GoldNPs were synthesized with the RITC-labeled EX-ABD-AFF, following the same protocol for synthesis of the EX-ABD-AFF-GoldNPs as described previously. The concentrations of the protein and dye were determined using the Bradford protein assay and fluorimetry, respectively.

For the PK study, C57BL/6 mice were injected with RITC-EX-ABD-AFF-GoldNPs via s.c. injection and blood samples were collected at 0, 0.5, 1, 2, 4, 8, 24, 48, 72, 96, 120, 144, 168, 192, 216, and 240 h post-administration. The collected blood was centrifuged at 5000 rpm for 5 min to obtain plasma samples, which were analyzed using a micro-plate reader (ex/em: 570/595 nm) [56]. PK profiles were analyzed by non-compartmental program (Phenix^®^ WinNonlin, Certara Inc., Princeton, NJ, USA) based on the plasma-concentration-versus-time data.

#### 3.6.5. Biodistribution

C57BL/6 mice were used for the administration of RITC-EX-ABD-AFF GoldNPs via s.c. injection. The mice were euthanized at 1, 4, and 7 days post-injection, and major organs were harvested. Fluorescent and brightfield images of the organs were captured using the VISQUE InVivo ART 400 (Vieworks, Anyang, Republic of Korea). The region of interest (ROI) for each organ was identified, and the intensity was measured.

### 3.7. Data Analysis

All the data were shown as the mean and the standard error of the mean. Statistical differences among the control vs. treatment groups were assessed using one-way ANOVA with the post hoc analysis using Tukey’s multiple comparison (Prism version 10.0, GraphPad, San Diego, CA, USA). Any result with *p*-value of less than 0.05 was considered statistically significant.

## 4. Conclusions

In this research, EX-ABD-AFF-GoldNPs were prepared by a one-step reduction process in an aqueous solution at ambient temperature. The physicochemical properties of the successfully prepared particles were measured by DLS, TEM/EDS, UV-VIS, and FT-IR. The EX-ABD-AFF-GoldNPs were stable in storage conditions sufficient for once-weekly preparation for the animal studies and exerted low cytotoxicity on the tested cell lines (Min6, HepG2, and B16F10) (up to 150 µg/mL, as with EX-ABD-AFF). In obese mice with a HFD diet, with subcutaneous injection, the EX-ABD-AFF-GoldNPs (50 nmol/kg) showed significant hypoglycemic activity, equivalent to that of the EX-ABD-AFF. Overall, this study demonstrated that the preparation of therapeutic protein-loaded gold NPs is feasible and, despite the much larger size compared with the protein, EX-ABD-AFF-GoldNPs can be successfully absorbed through the subcutaneous route and show nearly equivalent hypoglycemic activity to the EX-ABD-AFF protein. Finally, this study showed that long-lasting properties could be acquired by only coating EX-ABD-AFF onto gold NPs.

## Data Availability

All the data are available within this article.
